# Circular RNAs in glioma: Molecular functions and pathological implications

**DOI:** 10.1016/j.ncrna.2023.10.007

**Published:** 2023-10-25

**Authors:** Cheng Tang, Xinyi He, Lintao Jia, Xiao Zhang

**Affiliations:** Department of Biochemistry and Molecular Biology, Fourth Military Medical University, Xi'an, 710032, China

**Keywords:** Circular RNA (circRNA), Glioma, Diagnosis, Prognosis, Therapeutic targets

## Abstract

Circular RNAs (circRNAs) are a special class of non-coding RNAs with the ring structure. They are stable, abundant and conservative across mammals. The biogenesis and molecular properties of circRNAs are being elucidated, which exert regulatory functions not only through miRNA and protein sponge, but also via translation and exosomal interaction. Accumulating studies have demonstrated that circRNAs are aberrantly expressed in various diseases, especially in cancer. Glioma is one of the most common malignant cerebral neoplasms with poor prognosis. The accurate diagnosis and effective therapies of glioma have always been challenged, there is an urgent need for developing promising therapeutic intervention. Therefore, exploring novel biomarkers is crucial for diagnosis, treatment and prognosis of the glioma which can provide better assistance in guiding treatment. Recent findings found that circRNAs are systematically altered in glioma and may play critical roles in glioma tumorigenesis, proliferation, invasion and metastasis. Due to their distinct functional properties, they are considered as the potential therapeutic targets, diagnostic and prognostic biomarkers. This review elaborates on current advances towards the biogenesis, translation and interaction of circRNAs in many diseases and focused on the role of their involvement in glioma progression, highlighting the potential value of circRNAs in glioma.

## 1 Introduction

CircRNAs, one of non-coding RNAs, have become the latest research hotspot in recent years. Covalently closed circRNAs were originally recognized in plant viroid and Sendai virus in 1976, and then it was also identified in the transcripts of the human tumor suppressor gene DCC [1,2]. On account of their lack of 5′caps and 3′tails, circRNAs were previously recognized as by-products of spliceosome-mediated splicing errors with little biological functions [3]. However, in the decades of rapid development of biotechnology, there has been a wave of research into non-coding RNAs in the life sciences, and more than 183,000 circRNAs have been identified in the human genome [4]. Increasing research has shown that circRNAs possess diverse biological functions and are aberrantly expressed in a number of human diseases.

CircRNAs are widely distributed and highly stable across different species. Therefore, they are thought to have multiple potential biological functions. Considering the differential expression and regulation of circRNAs in different diseases, circRNAs are expected to serve as excellent biomarkers and promising therapeutic targets in clinical applications [5]. However, elucidating the exact mechanisms by which circRNAs participate in the progression of various diseases requires further research.

Glioma is one of the most common malignant cerebral tumors [6]. The incidence of glioma is about 8 per 100,000 population worldwide. And due to the high degree of invasiveness, median survival of high-grade glioma patients is only 15.4 months, and five-year survival is less than 5 % [7]. Conventional approaches for treating gliomas include surgery, chemotherapy and radiotherapy, which have achieved limited therapeutic effect [8]. Temozolomide (TMZ) is one of the most important first-line chemotherapy drug in glioma, especially in GBM. However, the disease becomes resistant to chemotherapy in up to 95 % of GBM patients after treatment [9]. Despite many studies on the mechanism of TMZ resistance, it has not been resolved. Therefore, new therapeutic strategies have been investigated. Recent studies have shown that immunotherapy has become potential therapeutic strategy which has been shown to be effective against glioma. As an illustration, locoregional delivery of HER2-specific CAR-T cells were shown to achieve curative efficacy in glioma patients in an ongoing phase I clinical trial [10]. Similarly, in another clinical trial, four out of six GBM patients treated with IL13Rα2-specific CAR-T cells showed improvement, providing a feasible treatment therapy for glioma [11]. In addition, numerous preclinical and clinical studies are performed to assess and explicit the efficacy of targeted therapy. With the advancement of sequencing technologies and computational algorithms, the properties and functions of circRNAs are becoming increasingly clear [12]. Recent findings found the differential expressions of circRNAs between glioma and para-carcinoma tissue, and demonstrated that they are involved in the regulation of glioma biological processes, highlighting the promising prospect of circRNAs as diagnostic and prognostic biomarkers. In the review, we summarized the involvement of circRNAs in glioma progression, paid special attention to the functional properties of exosomal circRNAs and the peptide or protein translated by circRNAs, and then explored their potential clinical applications for diagnosis, prognosis, and therapeutic targets in glioma. As the mystery of circRNAs are being unraveled, it is predictable that circRNAs will have promising application prospects.

## 2 CircRNA biogenesis

**Fig. 1:**
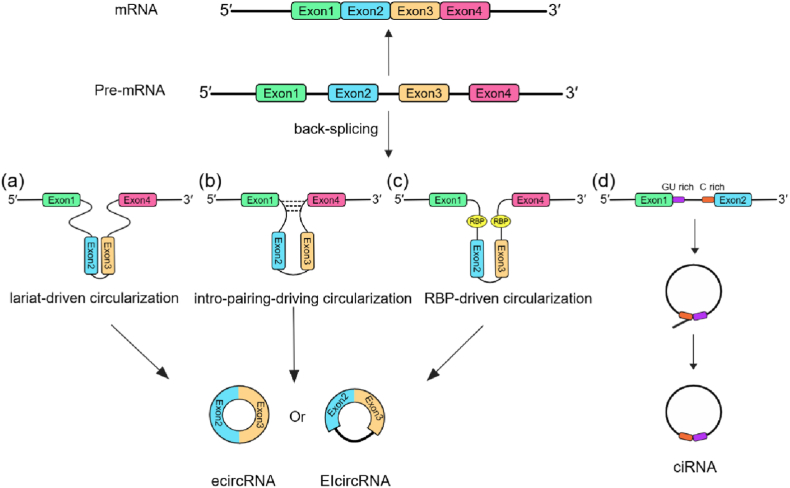
The biogenesis of circRNAs. Different from normal RNA splicing, the formation of circRNAs is based on back-splicing. (a) In lariat-driven circularization, the pre-mRNA produces a lasso by exon skipping, leading to the generation of EIcircRNAs and ecircRNA. (b) In intro-pairing-driving circularization, the complementary introns are paired to promote the formation of circRNAs. (c) In RBP-driven circularization, RBPs binding to the flanking introns at either end of the circular exon contain motifs contributes to circRNA biogenesis. (d) GU rich and C rich elements promote the formation of ciRNAs.

The formation of circRNAs differs from normal RNA splicing. Closed circular RNAs are mainly generated by back splicing, where the 5′ end site is ligated to the 3′ end site in a reverse direction [13]. CircRNAs mainly include exonic circRNAs (ecircRNAs), intronic circRNAs (ciRNAs) and exon-intron circRNAs (EIcircRNAs) according to the genomic source [[14], [15], [16]]. Exonic circRNAs are all originated from exons of maternal genes, whereas intronic circRNAs are originated from introns and depend on 7 nt GU rich and 11 nt C rich elements (Fig. 1) [15]. Significantly, EIcircRNAs are derived from both exons and introns and included three models as same as ecircRNAs: lariat-driven circularization, intro-pairing-driving circularization and RNA-binding proteins (RBP)-driven circularization (Fig. 1, a-c) [14].

**Fig. 2:**
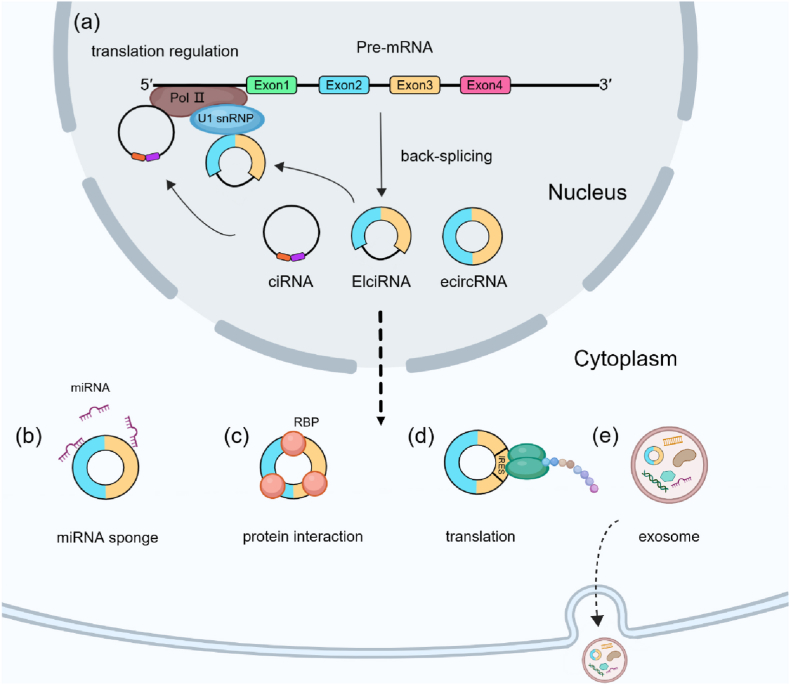
The biological regulatory mechanism of circRNAs. (a) Nuclear circRNAs can regulate transcription and splicing. (b) CircRNAs can act as miRNA sponges and regulate the expression of relevant genes. (c) CircRNAs can interact with RBPs to regulate these protein functions. (d) CircRNAs with IRES or m6A modification can be translated. (e) Exosomal circRNAs can serve as molecular biomarkers.

The regulation of circRNAs biogenesis involves a variety of modes (Fig. 2, a). RNA Pol II is known to affect pre-processing by controlling the transcription elongation rate, which is critical for the result of splicing [17]. The 5′splice site is tightly connected to the 3′ splice site by regulatory elements in introns flanking back spliced exons to stimulate circRNA biogenesis [18,19]. In addition, such RNA pairs can be derived from repetitive or non-repetitive elements [20]. With a length of about 300 nucleotides, circularization by Alu repeats may be relatively more complicated. Diverse RNA pairing and competitive IRAlus formation could be facilitated by the widespread Alu repeat elements in human introns to perform alternative circularization, suggesting their important role in alternative circularization in humans [19]. Although many regulatory mechanisms for circRNA biogenesis have been identified, the specific regulatory mechanism remains to be elucidated with an urgent need for further studies.

## 3 CircRNA interaction

### 3.1 miRNA sponge

CircRNAs are well suited to the role of miRNA sponges because of their stable circular structure and conserved sequences (Fig. 2, b). By binding miRNAs, the miRNAs’ target genes expression can be upregulated. The cerebellar degeneration-related protein 1 transcript (CDR1as) that could be combined with microRNA effector complexes contained more than 70 miR-7 target sites. As a miRNA sponge, CDR1as has a high binding capacity of miR-7 which is similar to the silencing of miR-7, and inhibits the development of the midbrain in zebrafish. Coincidentally, the testis-specific circRNA also played a sponge role in regulating miR-138 [21,22]. Significantly, most circRNAs contained fewer miRNA binding sites, demonstrating that they lack the ability to play the role of miRNA sponges.

### 3.2 Nuclear circular RNAs can regulate transcription and splicing

The location of circRNAs is mainly in the cytoplasm, while CiRNAs and EIciRNAs are found in the nucleus (Fig. 2, a) [14,16]. Ci-ankrd52 was shown to associate with Pol II elongation machinery and have a positive regulatory effect on Pol II transcription, indicating that non-coding intronic transcripts act in cis to regulate their parent coding genes [15]. Besides, the interaction of nuclear EIciRNAs and U1 snRNP can drive transcription to increase the expression of parental genes [16]. Although it is not clear how these circRNAs remain in the nucleus, there is evidence of their ability of regulating transcription and splicing.

### 3.3 Interact with RBPs

By interacting with related proteins to form circRNA-protein complexes, circRNAs can modulate protein functions (Fig. 2, c). According to the RBP sponge hypothesis, circCDYL with multiple RBP binding sites may regulate GRWD1 target genes by interacting with GRWD1 in HepG2 [23]. CircYap was found to be able to bind to Yap mRNA as well as to eIF4G and PABP, which play crucial roles in Yap translation, and the overexpression of circYap could repress Yap translation initiation [24]. Another study found that circVAMP3 binding to the complex of CAPRIN1 and G3BP1 promoted the formation of stress granule, and reduced the Myc proto-oncogene protein expression via promoting CAPRIN1 phase separation and inhibiting *c*-Myc translation, respectively [25]. Taken together, the interactions of circRNAs with RBPs may enrich their functions and contribute to many biological processes.

### 3.4 CircRNAs can be translated

Apart from the molecular functions and mechanisms mentioned above, there are also circular RNAs that have been found to be translatable in recent studies (Fig. 2, d). CircRNAs’ translation could be based on internal ribosome entry site (IRES) elements or m6A modification without 7-methylguanosine (m7G) cap and poly(A) tail [26,27].

The 40S subunits of ribosomes are recruited to RNAs by IRES elements to provide cap-independent protein translation initiation [28]. It is well known that engineered circRNAs containing an IRES can be translated. Most endogenous circRNAs do not associate with ribosomes, but several circRNAs with IRES have been proved to be translated [29]. For instance, circ-ZNF609 could be translated to a myoblast proliferation regulatory protein. In addition to IRES element-dependent pattern, m6A-driven translation of circRNAs was also observed. During mouse spermatogenesis, many circRNAs in late pachytene spermatocytes contained ORFs with m6A-modified start codons, which ensured stable protein production [30]. Besides, some research suggested that the translation of circRNAs could be completed by the rolling circle amplification approach. These circRNAs have only a start codon and no IRES or stop codon, and the number of nucleotides is a multiple of three. In theory, this could lead to the production of high-molecular-weight proteins as soon as translation begins [31]. Based on these studies, the translation of circRNAs has an effect on life activities and cell function, but the specific mechanisms of translation of circRNAs are not fully revealed.

### 3.5 Exosomal circRNAs

Exosomes are small extracellular vesicles about 30–150 nm in diameter that contains a diverse set of nucleic acids, proteins and lipids. Exosomes, secreted by various kinds of cells, circulate in body fluids and can transfer information to recipient cells (Fig. 2, e) [32]. More and more studies have shown that circRNAs are rich in exosomes and can function by exosomal interaction.

Exosomes protect circRNAs against enzymatic degradation due to the phospholipid bilayer membrane structure. Because of their high biocompatibility, circRNAs could cross the physiological barrier such as the blood-brain barrier (BBB) [33]. Circ-IARS promoted tumor progression by regulating HUVEC via exosomes delivery in pancreatic cancer [34]. Another study showed that circRNAs can be shed from cells into extracellular space, which is the main route by which cells clear circRNAs. In addition, the excreted circRNAs could lead to cell-to-cell communication through the uptake of exosomes by other cells [35]. For example, the ferroptosis of LUAD cells will be desensitized by the upregulation of FABP3 and then the downregulation of AA in a FABP3-dependent manner due to the upregulation of circRNA_101093 via exosomes secreted by tumor cells [36]. However, additional research is needed to elucidate the functional and molecular mechanisms of exosomal circRNA to open new avenues of insight and provide novel therapeutic approaches in various diseases (Fig. 2).

## 4 CircRNA detection

Since circRNAs are mainly derived from exons of RNA, the key to distinguish and detect circRNAs is to identify the back-splicing junction (BSJ) [37]. A series of experimental techniques have been used to detect circRNAs. RNase R is a type of *Escherichia coli* exonuclease, which can cleave and degrade RNA in the 3′ to 5’ direction. However, circRNAs are not affected by RNase R digestion due to their stable structures compared with linear RNA molecules, so RNase R digestion experiment can be adapted for circRNA enrichment [38]. Enrichment of circRNA can improve the detection of BSJ in circRNA. Northern blotting, the gold standard approach for circRNA analysis, is typically combined with RNase R digestion to detect circRNAs by employing a hybridization probe paired with the BSJ. But it is noteworthy that Northern blotting is complex and time-consuming [39]. Another common experimental approach for circRNA detection is RT-qPCR. It has been widely used in the detection, verification and even quantification of circRNAs with the advantage of time-saving and sensitive. However, due to the complexity of circRNAs structures, quantification of RT-qPCR may be inaccurate. Then, the researchers improve the specificity of PCR amplification effectively and achieve the purpose of accurate detection and quantification of circRNA by designing different primers according to the sequence of the BSJ [40].

Besides these experimental approaches mentioned above, RNA-seq is also widely applied in the field of circRNA identification and detection. The second-generation sequencing is the most widely used technology for circRNAs identification and the full-length transcriptome sequencing of rRNA-deleted database is particularly popular. The second-generation sequencing database construction methods include rRNA-deleted library and rRNA-deleted, RNase R+ library [41]. After sequencing, a series of bioinformatics analysis can be performed. However, there are also some shortcomings in the detection and quantification of circRNA by second-generation sequencing. For example, the fragmentation of RNA molecules by second-generation sequencing may lead to the loss of the BSJ. And the expressions of some circRNAs are not high, making their signals in the RNA library easily masked [42]. The third-generation sequencing of circRNA can easily detect the information that is difficult to capture by the second-generation sequencing. It not only supports the diversity of circRNA species, but also reveals the complexity of the internal sequence of BSJ. At present, the strategies of using nanopore sequencing technology to detect full-length RNA include iosCirc and CIRI-long, etc [43,44]. However, there are some problems with third-generation sequencing. For example, the sequencing depth is not sufficient, it is difficult to calculate the expression of relative linear molecules [45]. Generally speaking, different detection techniques have their own advantages, and different methods can be selected according to specific requirements.

## 5 CircRNA and diseases

### 5.1 CircRNA and cardio-cerebral vascular diseases

A growing body of evidence suggests circRNAs are involved in several human diseases, such as the cardiovascular diseases. One study identified a set of 575 candidate circRNAs that are derived from genes that are associated with cardiovascular diseases [46]. In the coronary artery disease study, 24 differentially expressed circRNAs were identified and 9 of them were shown to upregulate the expression of TRPM3 via sponging hsa-miR-130a-3p [47]. These studies inspire us that the in-depth exploration of circRNA will revolutionize cardiovascular diseases diagnosis and therapy. In addition to cardiovascular disease, circRNAs are implicated in cerebrovascular diseases. For example, circSCMH1 was found to be decreased in patients with acute ischemic stroke and to have the ability to conduct the enhancement of the neuronal plasticity and the inhibition of glial activation [48]. CircSHOC2 was found to suppress neuronal apoptosis and ameliorate neuronal damage by sponging miR-7670–3p [49]. In another study, the overexpression of circOGDH inhibited neuronal cell viability in patients with acute stroke, showing the potential of acting as a biomarker [50].

### 5.2 CircRNA and chronic diseases

CircRNAs have been proved to be involved in autoimmune diseases, such as systemic lupus erythematosus (SLE), which can cause multi-system damage and have a major impact on human health. PKR phosphorylation was increased and circRNA expression was decreased in peripheral blood mononuclear cells in SLE patients [51]. Several circRNAs are also associated with the pathogenesis of rheumatoid arthritis (RA). For instance, circRNA_09505 sponged miR-6089 and regulated RA via miR-6089/AKT1/NF-κB axis in CIA mice [52]. Similarly, diabetes, as a typical chronic disease that also seriously affects people's health, is also closely related to circRNAs. The overexpression of circPPM1F led to the exacerbation of pancreatic islet injury by activating M1 macrophage. It showed the potential of targeting circPPM1F to be a novel therapeutic approach for type 1 diabetes among children [53]. In the area of chronic inflammation, knockdown of circFOXO3 was reported to protect against cigarette smoke-induced inflammation, regulated by downregulation of IKK-β mRNA through sponging of miR-214-3p and suppression of the NF-κB pathway [54]. In osteoarthritis (OA), circRNA.33186 was found to be associated with the proliferation and apoptosis of chondrocytes and play important roles in promoting the progression of OA by serving as miR-127–5p sponge [55]. In another study, restoring circBNC2 was able to inhibit epithelial cell G2-M arrest which contributed to the prevention of epithelial organ fibrosis [56].

### 5.3 CircRNA and cancers

It is striking that circRNAs are differentially expressed among different types of cancer and have an effect on oncogenesis and cancer progression. CircRNAs have different expression patterns in different cancers. Although the mechanisms of the aberrant expression of circRNAs in cancers remain unclear, circRNAs have been confirmed to be have various roles among different cancers. CircRNAs may function in cancer by regulating the tumor development such as cell proliferation, apoptosis, migration and invasion [57]. For example, has_circRNA_104348 can promote HCC cell proliferation, invasion and migration, and suppress cell apoptosis by regulating miR-187–3p/RTKN2 signaling pathway and activating Wnt/β-catenin pathway [58]. In another research, circBCAR3 was found to facilitate the proliferation, migration and invasion of esophageal cancer cells by binding with miR-27a-3p [59]. Meanwhile, circRNAs have also been found to be involved in tumor angiogenesis and tumor immunity. Recent evidence showed that circFOXP1 could contribute to tumor angiogenesis through miR-127–5p/CDKN2AIP axis in osteosarcoma [60]. And circNDUFB2 was able to induce the immune responses in non-small lung cancer by regulating the degradation of IGF2BPs, which provided a novel target of immunotherapy [61]. Due to their specific expression and distinct biological functions in cancer progression and outcome, circRNAs have important clinical implications. CircRNAs can potentially act not only as diagnosis and prognosis biomarkers, but also as promising therapeutic targets. For example, HCC patients with low circVAMP3 expression have poor prognosis and circVAMP3 is significantly downregulated in HCC tissues, suggesting that circVAMP3 is a potential prognostic indicator for HCC and may serve as a therapeutic target for HCC treatment [62]. While in glioma, the effect of circRNA-0002109 has been proved to be related to the promotion of glioma malignant progression [63]. Next, this review will further clarify the functions of circRNAs by describing their involvement in glioma.

## 6 CircRNA and glioma

### 6.1 Expression profile

Although the circRNA expression profile in glioma remains unclear, the results of existing studies indicate that aberrant expression of circRNAs correlates with glioma tumorigenesis and progression. The expression profiles of circRNAs between glioma and non-tumorous adjacent samples were identified using microarray technology, and the data indicated that 91 circRNAs were differentially expressed in glioma tissues compared to non-tumorous adjacent tissues. Among these, the upregulated circ_0037655 was highlighted and found to have the ability to induce tumor cell survival and invasion. In addition, high-throughput RNA-seq was performed to analyze 5 glioma tissues and normal brain tissues, and 20,204 circRNAs were sequenced to perform differential analyses [64]. Furthermore, most circRNAs were found downregulated in the identification of 1411 differentially expressed circRNAs after sequencing circRNAs in 5 GBM and 5 normal brain samples [65]. In addition, a bioanalysis of downregulated circRNAs during neurogenesis was performed, and 9 circRNAs in glioma samples was examined. In the follow-up study, the overexpression of circ-MAPK4 was found in early neurodevelopment and glioma tissues, indicating that a cancer-promoting effect of circ-MAPK4 in glioma [66]. In another study, 2289 differentially expressed circRNAs were identified by analyzing 12 pairs of GBM samples and adjacent normal brain samples. Among them, the differential expression level of circular *E*-cadherin RNA (circ-E-Cad) was the most significant and was shown to promote glioblastoma tumorigenicity [67]. The aberrant expression of circRNAs shows that circRNAs hold promise as biomarkers in oncogenic or tumor suppressor studies of glioma by identifying and analyzing different specific circRNAs.

### 6.2 Oncogenic

Based on recent studies, some circRNAs have been found to have a positive effect on glioma progression. Compared to normal cells, cancer cells are characterized by rapid and unrestricted proliferation. A huge body of research has shown that circRNAs can maintain the proliferative signal of glioma cells. A circular RNA-encoded oncogenic *E*-cadherin variant is essential for the maintenance of the oncogenicity for glioma stem cells by activating EGFR independently of EGF via binding to the EGFR CR2 domain [67]. In addition, the expression of circCPA4 was high in glioma and played the role of miR-760 sponge to indirectly regulate MEF2D. Knockdown of circCPA4 suppressed tumor cell growth, showing a positive role of circCPA4 in malignant cell phenotypes [68]. Significantly, circRNAs can also help suppress or prevent glioma cell apoptosis. For example, circRNA MAPK4 inhibited cell apoptosis via the p38/MAPK axis by playing the sponge role for miR-125a-3p in glioma [66]. Another research indicated that the expression of circRNA PIP5K1A was higher in glioma samples than that in normal samples and TCF12 and PI3K/AKT were activated through sponging miR-515–5p by circRNA PIP5K1A. Due to the regulation of circRNA PIP5K1A, the malignant behaviors of glioma was exacerbated and apoptosis was induced [69].

Invasion and metastasis occur when tumors grow to a large size, and tumor cells can no longer acquire essential nutrients from surrounding cells and the environment. As the main malignant behaviors of cancer cells, invasion and metastasis can also be promoted by circRNAs. EIF4A3-induced circMMP9 was able to accelerate GBM cell proliferation as well as invasion and metastasis by sponging miR-124 [70]. Coincidentally, a feedback loop was found in glioma. Circ-RPL15 stimulated proliferation and migration of gliomas by sponging miR-146b-3p and then upregulating VEGFA [71]. What's more, circGLIS3 upregulation promoted glioma cell migration, invasion and invasiveness in tumor-bearing mice by promoting Ezrin T567 phosphorylation [72].

In addition to proliferation, invasion and metastasis, sustained angiogenesis is an important hallmark of cancer. Angiogenesis is the process through which new blood vessels develop from the pre-existing vessels and is believed to be a critical factor in tumor cell viability and proliferation [73]. Recent evidences showed that circRNAs can increase the angiogenic capacity of glioma. Circ-ATXN1 served as a miR-526b-3p sponge to inhibit the function of miR-526b-3p to downregulate MMP2/VEGFA expression, leading to glioma angiogenesis [74]. Furthermore, circ-DICER1 binding to MOV10 promoted angiogenesis by sponging miR-103a-3p or miR-382–5p to prevent the downregulation of ZIC4 production. And then the expression of hsp90β was increased by ZIC4 to activate the PI3K/Akt axis, leading to the promotion of glioma angiogenesis [75].

As one of key members of the immunosuppressive microenvironment of gliomas, glioma-associated immune cells make a critical contribution to tumor development and immunosuppressive properties [76]. CircNEIL3 was able to protect the cancer-promoting protein IGF2BP3 from ubiquitination mediated by HECTD4. In addition, tumor cells with high circNEIL3 expression drove macrophage infiltration into the tumor microenvironment via delivering exosomes containing circNEIL-3 to tumor-associated macrophages (TAMs) and then acquired immunosuppressive properties via IGF2BP3 [77]. In addition, as a critical part of tumorigenesis and progression, enhanced energy metabolism is able to provide necessary nutrients to tumor cells, which has been shown to be modulated by circRNAs. Serving as a miR-378e sponge, circNFIX positively regulated RPN2 to promote glucose metabolism in glioma cells [78]. Furthermore, through the miR-361–5p/TPX2 signaling pathway, highly expressed circPOSTN participated regulating TPX2 expression in glioma cells, resulting in enhanced proliferation and aerobic glycolysis [79]. Overall, circRNAs play an oncogenic role in glioma development, showing a novel perspective for understanding glioma biology and offering their great potential for targeted therapies.

### 6.3 Tumor suppressor

On the contrary, some circRNAs can also play tumor suppressor roles in glioma. However, the number of studies on tumor suppressor circRNAs in glioma is less than that of oncogenic circRNAs. Compared to other normal organ tissues, the CDR1as expression was markedly higher in the brain. However, the CDR1as expression was downregulated in glioma compared to normal brain tissues. Notably, CDR1as was found to have an inhibitory function on tumorigenesis by destabilizing the p53/MDM2 complex, through which MDM2-mediated ubiquitination was decreased to stabilize p53 by CDR1as [80]. In another research, circ_SFMBT2 played a negative role in glioma cells and could abolish the influence of mir-182-5p-induced tumor development in glioma by sponging mir-182–5p [81]. In addition, the circBRAF expression was downregulated in glioma, and it could suppress malignant behaviors of glioma via regulating the miR-1290/FBXW7 axis [82]. And the expression of hsa_circ_0001017 was also downregulated in glioma samples, while high levels of hsa_circ_0001017 expression led to tumor inhibition via regulation of the let-7g-3p/NDST3 signaling pathway [83].

**Fig. 3:**
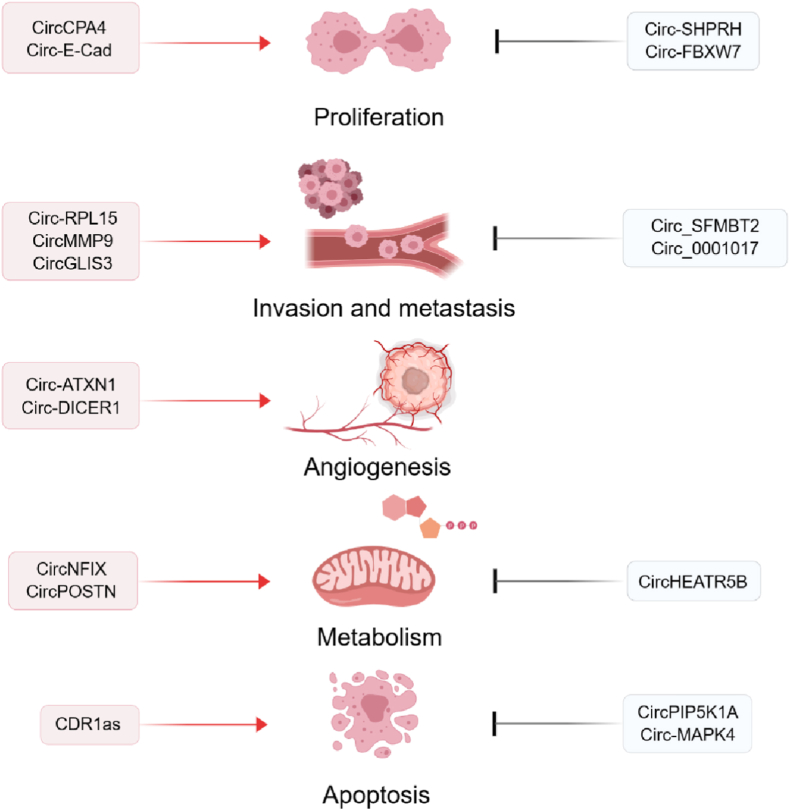
The functions of circRNAs in the malignant phenotype of glioma. In glioma, circRNAs affect cell proliferation, invasion, metabolism and apoptosis, tumor metastasis and angiogenesis.

In addition to interacting with proteins or functioning as miRNA sponges, circRNAs can also be translated and inhibit glioma cell progression. Recent evidence showed that circular SHPRH (circ-SHPRH) can translate a 17kDA protein SHPRH-146aa. SHPRH-146aa protected SHPRH from ubiquitin-mediated proteasomal degradation and acted as an E3 ligase, resulting in suppression of glioma tumorigenesis and proliferation [84]. Aerobic glycolysis was a metabolic hallmark of GBM, but the role of circRNAs in the process remained unclear. One study showed that aerobic glycolysis can be inhibited by the protein encoded by ZCRB1-induced circHEATR5B in GBM via phosphorylation [85]. In another example, circ-FBXW7, which possesses the spanning junction open reading frame, was able to encode FBXW7-185aa. It can inhibit cell proliferation in glioma, whereas the knockdown of FBXW7-185aa stimulated glioma progression (Table 1) [86]. Because of the important biological significance in glioma, the tumor suppressor role of circRNAs also provides an optional approach in glioma therapy (Fig. 3).

**Table 1:** Representative circRNAs and corresponding signaling pathways in glioma.

Name	Expression status in glioma	Possible mechanism	Function*	Clinical significance	References
Circ-MAPK4	up	Circ-MAPK4/miR-125a-3p/p38/MAPK	Proliferation (+) Apoptosis (−)	Therapeutic target	[66]
Circular *E*-cadherin (circ-E-Cad) RNA	up	Circular *E*-cadherin (circ-E-Cad) RNA/EGFR–STAT3	Tumorigenesis (+)	Therapeutic target	[67]
CircCPA4	up	CircCPA4/miR-760/MEF2D	Proliferation (+) Migration (+) Invasion (+)	Therapeutic target	[68]
CircPIP5K1 A	up	CircPIP5K1A/miR-515–5p/TCF12/PI3K/AKT	Proliferation (+) Invasion (+) Apoptosis (−)	Prognosis	[69]
CircMMP9	up	CircMMP9/miR-124	Proliferation (+) Invasion (+) Metatasis (+)	Therapeutic target	[70]
Circ-RPL15	up	Circ-RPL15/miR-146 b-3p/VEGFA	Proliferation (+) Invasion (+) Migration (+)		[71]
CircGLIS3	up	CircGLIS3/Ezrin	Invasion (+) Migration (+) Angiogenesis (+)		[72]
Circ-ATXN1	up	SRSF10/circ-ATXN1/miR-526 b-3p	Migration (+) Angiogenesis (+)	Therapeutic target	[74]
Circ-DICER1	up	MOV10/circ-DICER1/miR-103a-3p (miR-382–5p)/ZIC	Migration (+) Angiogenesis (+)	Therapeutic target	[75]
CircNEIL3	up	CircNEIL3/HECTD4/IGF2BP3	Immunosuppressive properties (+)	Prognosis, therapeutic target	[77]
CircNFIX	up	CircNFIX/miR-378e/RPN2	Migration (+) Invasion (+) Metabolism (+)		[78]
CircPOSTN	up	CircPOSTN/miR-361–5p/TPX2	Apoptosis (−) Metabolism (+)		[79]
CDR1as	down	CDR1as/p53/MDM2	Proliferation (−) Tumorigenesis (−) Apoptosis (+)	Therapeutic target	[80]
Circ_SFMBT2	down	Circ_SFMBT2/miRNA-182–5p/metastasis suppressor 1	Proliferation (−) Migration (−) Invasion (−)	Therapeutic target	[81]
CircBRAF	down	CircBRAF/miR-1290/FBXW7	Proliferation (−) Metastasis (−)		[82]
Hsa_circ_0001017	down	Hsa_circ_0001017/let-7g-3p/NDST3	Proliferation (−) Metastasis (−) Apoptosis (+)		[83]
Circ-SHPRH	down	Circ-SHPRH/SHPRH-146aa	Proliferation (−) Tumorigenesis (−)		[84]
CircHEATR5B	down	ZCRB1/circHEATR5B/HEATR5B-881aa/JMJD5/PKM2	Proliferation (−) Metabolism (−)	Therapeutic target	[85]
Circ-FBXW7	down	Circ-FBXW7/FBXW7-185aa	Proliferation (−)	Prognosis	[86]

*. (+) represents promotion, (−) represents inhibition.

### 6.4 Therapeutic targets

Numerous studies show that circRNAs have distinct functions and play diverse roles in glioma, suggesting their possibility as therapeutic targets for glioma therapy. Considering that circRNAs significantly promote cell proliferation, invasion or tumor metastasis, it is promising to treat glioma by suppressing the expression of these circRNAs. For example, circHECTD1 is critical for glioma proliferation and invasion, and knockdown of circHECTD1 suppressed glioma growth in a mouse xenograft model. It was shown that circHECTD1 expression could be downregulated to intervene in glioma progression as a novel therapeutic target [87].

As conventional therapies for glioma have limited therapeutic effect due to the characteristics of glioma cells, the therapeutic resistance of which is a major difficulty in glioma treatment. EIF4A3-induced circASAP1 promoted NRAS expression through miR-502–5p sponging, which led to glioma cell proliferation and temozolomide resistance. Due to its regulatory functions, circASAP1 can act as a potential interference target, and ceRNA-mediated microRNA sequestration may become an effective therapeutic strategy for glioma treatment [88]. Besides, circRNAs are also found to function as important regulators in glioma radiation resistance. A study indicated that the circ_0008344 expression was markedly higher in glioma with resistance to radiotherapy and subsequent experiment proved that the decrease of circ_0008344 inhibited glioma progression and enhanced the radiosensitivity in glioma by acting on miR-433–3p/RNF2 axis [89]. In another study, circ_VCAN regulated the radio-resistance of glioma by acting as the miR-1183 sponge [90]. These evidences indicated that circRNAs hold promise as therapeutic targets in glioma radiotherapy.

One promising strategy is immunotherapy, which can penetrate the blood-brain barrier in glioma and has been highlighted in abundant research [8]. Exosomal circRNAs can act as tumor antigens to induce active antitumor immunity. PD-L1 was involved in tumor immunotherapy, miR-34a inhibited glioma development by targeting PD-L1, suggesting the therapeutic potential of miR-34a. And through the Notch signaling pathway, circNFIX could downregulate miR-34a-5p in glioma, providing not only a novel insight into the cancer-promoting effect of circNFIX, but also a potential therapeutic target in glioma immunotherapy [91]. By targeting immune checkpoints PD-1 and CTLA-4, miR-138 can play glioma suppressor roles. However, circ_002136 could sponge miR-138–5p, indicating that circ_002136 is a potential therapeutic target [92,93].

The regulatory functions of circRNAs in glioma offer a novel approach to improve efficacy and stability. However, clinical trials have achieved limited improvements, which indicates the necessity of further studies to resolve difficulty in glioma therapy.

### 6.5 Diagnosis and prognosis

Given the difficulty of differential diagnosis with other similar brain lesions and the invisibility of early glioma, there are still a number of challenges in improving the efficiency of glioma diagnosis. In addition, the prognosis of glioma patients is not ideal, indicating that more efforts are needed to improve patient survival. Considering their stable structure and aberrant expression in glioma, circRNAs hold promise as diagnostic and prognostic biomarkers in glioma. It is reported that the poor prognosis of glioma patients was highly linked with the upregulation of circ-CDC45 in glioma samples, because circ-CDC45 had the oncogenic functions of promoting glioma progression. The study demonstrated the great potential of circ-CDC45 as a diagnosis and prognosis biomarker [94]. Another study showed that the circ_0034642 expression was markedly high in glioma and contributed to hypoxia-induced glycolysis and malignant phenotypes in glioma, providing a novel approach for glioma diagnosis [95].

The conventional diagnosis scheme of glioma is nuclear magnetic resonance [96]. However, it is often not scheduled as part of a general physical examination because of its high cost. And due to its concealed onset, glioma has progressed to the middle to late stage once diagnosed, which also results in poor survival in glioma patients [97]. Therefore, the early diagnosis of glioma is particularly important. Liquid biopsy holds great promise for glioma early diagnosis because it has the characteristics of non-invasive, sensitive and dynamic. CircRNAs are highly stable, often expressed in specific ways in tissues and developmental stages, and is abundant not only in cells and tissues but also in body fluids, making circRNAs an ideal candidate for biomarkers of glioma liquid biopsy [98]. For example, by collecting cerebrospinal fluid samples from medulloblastoma patients and comparing them to normal cerebrospinal fluid, circ_463 was found to be differentially expressed and had the potential to be used as a novel biomarker [99]. Besides, circ_0005198 was able to enhance TMZ resistance and was proved to be upregulated in serum samples from glioma patients compared with healthy controls, indicating the potential for circ_0005198 to act as a therapeutic target [100]. In addition to the direct detection of free circRNAs in body fluids, recent studies have shown that exosomal circRNAs in body fluids is abundant and more stable, demonstrating the great potential of disease monitoring through detecting them. As a result, more and more attention has been paid to exosomal circRNAs in liquid biopsy.

**Fig. 4:**
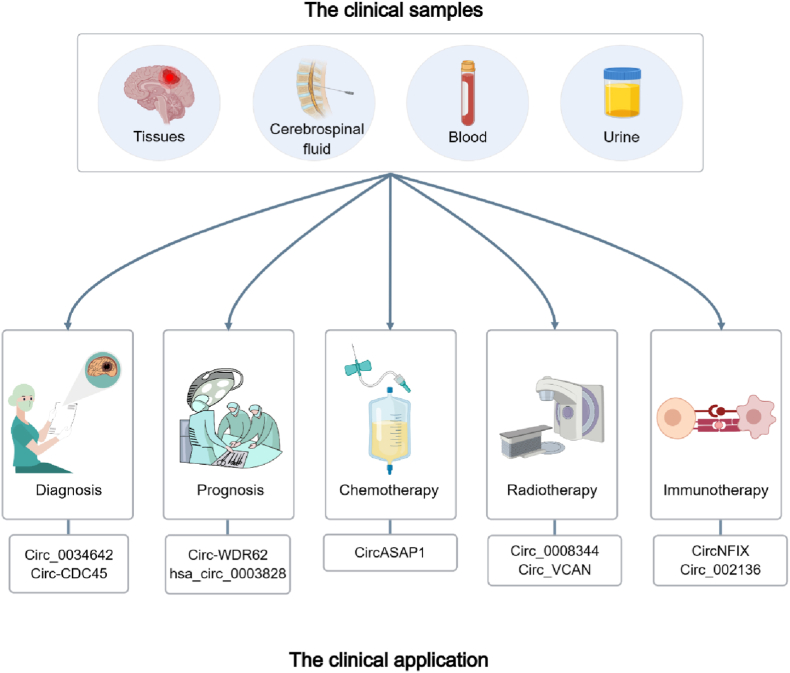
The clinical significance of circRNAs in glioma. CircRNAs are typically extracted from clinical samples such as tumor tissues, cerebrospinal fluid, blood and urine. CircRNAs are expected to play important roles in the diagnosis, prognosis and therapy of glioma.

Exosomes, as a cargo for cell-to-cell communication, transport molecules into the environment that alter or reprogram the tumor microenvironment. Significantly, due to their abundance in exosomes and detectability in the circulation, exosomal circRNAs are considered to have high clinical value in the diagnosis and prognosis of glioma [101]. For example, exosomal circWDR62 regulated the MGMT expression by serving as a miR-370–3p sponge, leading to the promotion of temozolomide resistance and malignant progression. It demonstrated that serum exosomal circWDR62 is expected to be a promising therapeutic target and prognosis biomarker in glioma [102]. Moreover, serum exosomal circRNAs hsa_circ_0075828, hsa_circ_0003828 and hsa_circ_0002976 were identified in astrocytoma. These circRNAs could be used to discriminate cancer patients from the healthy, demonstrating their potential as measurable indicators for glioma prognosis (Fig. 4) [103].

Overall, the application of circRNAs in the diagnosis and prognosis of glioma is promising due to their stable structures, multiple functions and wide distribution in the circulation.

CircRNAs are typically extracted from clinical samples such as tumor tissues, cerebrospinal fluid, blood and urine. CircRNAs are expected to play important roles in the diagnosis, prognosis and therapy of glioma.

## 7 Conclusion

Given few strategies available for glioma treatment and poor prognosis, there is an urgent and pressing need for developing new ideal management for early diagnosis and treatment in patients. Considering the unique loop structure, circRNAs are abundant and play unique roles in biological processes. Their expression level is basically high in the brain and changes significantly during the development of the nervous system or the occurrence of neurological disorders [104]. A lot of research has proved that circRNAs have a critical effect on glioma progression and tumorigenicity, even via exosomal interaction. Exosome can pass blood-brain barrier and released into the bloodstream. The exosomal circRNAs will be potential biomarkers for early diagnosis of glioma using liquid biopsy. What's more, their abundant biological functions provide promising prospect for glioma targeting therapy.

However, there are currently no circRNAs used in clinical trials. This may be due to the high cost and difficulty in purification for targeted therapy and limitations of detection technologies for clinical diagnosis. Although many research will be required to make them available in clinic, we believe circRNAs will achieve their great potential in clinical application, providing a novel approach to fight against glioma.

## Funding

This work was supported by the 10.13039/501100001809National Natural Science Foundation of China (No. 81903149 and 82172910) and the 10.13039/501100007128Natural Science Foundation of Shaanxi Province, China (No. 2021SF-058).

## Declarations of interest

None.

## CRediT authorship contribution statement

**Cheng Tang:** Investigation, Methodology, Project administration, Writing – original draft. **Xinyi He:** Investigation, Methodology, Writing – original draft. **Lintao Jia:** Funding acquisition, Supervision, Validation, Writing - review & editing. **Xiao Zhang:** Conceptualization, Funding acquisition, Project administration, Validation, Writing - review & editing.

## Abbreviations List

ecircRNAs: exonic circRNAs
ciRNAs: intronic circRNAs
EIcircRNAs: exon-intron circRNAs
IRES: internal ribosome entry site
m7G: 7-methylguanosine
BBB: blood-brain barrier
SLE: systemic lupus erythematosus
RA: rheumatoid arthritis
TAMs: tumor-associated macrophages
